# Long noncoding RNA plasmacytoma variant translocation gene 1 promotes epithelial‐mesenchymal transition in osteosarcoma

**DOI:** 10.1002/jcla.23587

**Published:** 2020-09-22

**Authors:** Chuanhui Xun, Dawei Jiang, Zheng Tian, Akbar Yunus, Jiangtao Chen

**Affiliations:** ^1^ Department of Orthopedics The First Affiliated Hospital of Xinjiang Medical University Urumqi China

**Keywords:** EMT, lncRNA PVT1, osteosarcoma, prognosis

## Abstract

**Objective:**

Long noncoding RNAs (lncRNAs) are involved in the proliferation, migration, and invasion of tumors. In the current study, our aim was to explore the role of lncRNA plasmacytoma variant translocation gene 1 (PVT1) in osteosarcoma.

**Methods:**

Quantitative real‐time reverse transcription‐polymerase chain reaction was used to detect the expression of lncRNA PVT1 in osteosarcoma tissues and cells. The relationship between lncRNA PVT1 expression status and the prognosis of patients with osteosarcoma was analyzed. The effect of lncRNA PVT1 on the malignant biological behavior of osteosarcoma cells in vitro was also analyzed.

**Results:**

LncRNA PVT1 was upregulated in osteosarcoma. High lncRNA PVT1 expression indicated poor prognosis in patients with osteosarcoma. In vitro knockdown of lncRNA PVT1 inhibited the proliferation, migration, and invasion ability of osteosarcoma cells. In addition, we confirmed that lncRNA PVT1 affected the epithelial‐mesenchymal transition of osteosarcoma cells.

**Conclusion:**

LncRNA PVT1 is a potential therapeutic target for osteosarcoma.

## Graphical abstract

LncRNA plasmacytoma variant translocation gene 1 (PVT1) promotes epithelial‐mesenchymal transition, proliferation, migration, and invasion of osteosarcoma cells.

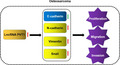

## 1 INTRODUCTION

Osteosarcoma is a common type of malignant bone tumor in adolescents, accounting for 5%‐10% of all malignancies among adolescents. Osteosarcoma is the sixth most common cause of cancer‐related deaths in adolescents.^1^ The main cause of death in osteosarcoma is lung metastasis. The 5‐year survival rate of patients with osteosarcoma is below 20%, even after active treatment.^2^ Targeted therapy and immunotherapy have improved the treatment of malignant tumors. However, there are limited effective therapeutic targets for osteosarcoma. Therefore, there is a need to elucidate the molecular mechanism underlying the progression of osteosarcoma and identify new therapeutic targets for osteosarcoma to enhance prognosis.

Long noncoding RNAs (lncRNAs) are involved in the proliferation, migration, and invasion of tumors.^3^ Recent studies have revealed that some lncRNAs such as lncRNA BC040587, lncRNA TUSC7, and lncRNA MALAT1, which are differentially expressed in osteosarcoma, are involved in the progression of osteosarcoma.^4^ LncRNA plasmacytoma variant translocation gene 1 (PVT1), which is localized on chromosome 8q24.21,^5^ is reported to be a Myc activator in plasmacytoma.^6^ Some studies have reported that the expression of lncRNA PVT1 is aberrant in different malignant tumors, including breast, gastric, and colorectal cancers.^7^, ^8^, ^9^ LncRNA PVT1 can promote the malignant phenotype of tumor cells by regulating DNA rearrangement and interacting with other oncogenes.^10^ However, the role of lncRNA PVT1 in osteosarcoma has been evaluated in only a limited number of studies.

Epithelial‐mesenchymal transition (EMT) of cells is known to play an indispensable role in the progression of tumors. EMT is a reversible cellular process that promotes invasiveness and is traditionally believed to be the prelude to the process of metastasis, wherein cells within a primary tumor lose their epithelial characteristics and acquire both the phenotype and a transcriptional program reminiscent of mesenchymal cells.^11^, ^12^ Multiple studies have shown that lncRNAs are involved in the regulation of EMT in osteosarcoma cells. The expression of lncRNA PGM5‐AS1 is upregulated in osteosarcoma. It regulates the expression of miR‐140‐5p and FBN1 through sponge adsorption, thereby promoting tumor EMT, invasion, and metastasis.^13^ LncRNA CCAT2, a marker of poor prognosis in patients with osteosarcoma, was found to promote EMT in osteosarcoma cells.^14^ Therefore, we also studied the relationship between lncRNA PVT1 and EMT in osteosarcoma cells.

Here, we investigated the expression and molecular function of lncRNA PVT1 in osteosarcoma. The findings of this study suggest that lncRNA PVT1 is a potential therapeutic target for osteosarcoma.

## 2 METHODS

### 2.1 Bioinformatics analysis

The GEO2R online analysis tool (https://www.ncbi.nlm.nih.gov/geo/geo2r/) was employed to examine five GeneChip expression microarrays of osteosarcoma (GSM954792, GSM954810, GSM954823, GSM954797, and GSM954825) to screen differentially expressed lncRNAs. The threshold for differentially expressed lncRNAs was set as follows: log_2_ (fold change) >2; adjusted *P*‐value < .05. The differentially expressed lncRNA results from the five datasets were visualized on a volcano plot. The Gene Expression Profiling Interactive Analysis (GEPIA) online tool (http://gepia.cancer‐pku.cn) was utilized to determine the expression of differentially expressed genes in osteosarcoma and their correlation with patient prognosis.

### 2.2 Specimen collection

This study was approved by the Ethics Committee of the First Affiliated Hospital of Xinjiang Medical University. In total, 78 pairs of surgically resected tumor and adjacent non‐tumorous tissues were collected between January 2015 and December 2018. The resected tissue was snap‐frozen in liquid nitrogen and stored at 80°C. All patients were diagnosed with osteosarcoma based on pathological analysis. The patients did not undergo chemotherapy, radiation therapy, or targeted therapy before surgery. All patients provided written informed consent to use the tissue specimens for the study.

### 2.3 Cell culture and construction of stable cell lines

The osteosarcoma cell lines (MG‐63, SW1353, Saos‐2, and U2OS) and a human osteoblast cell line (hFOB1.19) were purchased from the American Type Culture Collection (ATCC). The cells were cultured in Dulbecco's modified Eagle's medium (DMEM; HyClone) supplemented with 10% fetal bovine serum (FBS; Thermo Fisher Scientific) and 1% penicillin (Biosharp) at 37°C and 5% CO_2_. The lentiviral vector containing the lncRNA PVT1 shRNA (shPVT1#1:5'‐CCCAACAGGAGGACAGCUUTT‐3'; shPVT1#2:5'‐GGACTTGAGAACTGTCCTTAC‐3'; shPVT1#3:5'‐GCTCCACCCAGAAGCAATTCA‐3') was designed and synthesized by GENECHEM Biotech (http://genechem.bioon.com.cn/). The cells were cultured until the logarithmic phase and transfected with the lentiviral vector containing green fluorescent protein (GFP) (multiplicity of infection = 30). The empty virus vector was used as the negative control (shNC). The transfected cells were cultured in the presence of puromycin (Solarbio) for 5 days to screen for stable cell lines with low lncRNA PVT1 expression. The efficiency of lncRNA PVT1 knockdown was confirmed by quantitative real‐time reverse transcription‐polymerase chain reaction (qRT‐PCR).

### 2.4 RNA extraction and qRT‐PCR

Total RNA was extracted from the cells or tissues using TRIzol reagent (Invitrogen). The purity of the extracted RNA was measured using a spectrophotometer (Unico). Next, the extracted RNA was subjected to reverse transcription to obtain cDNA using the PrimeScript RT‐PCR kit (TaKaRa). qRT‐PCR analysis was performed using SYBR™ Premix Ex Taq™ (TaKaRa) in the 7500 Fast Real‐Time System (Applied Biosystems). *GAPDH* was used as a loading control. The relative expression of lncRNA PVT1 was calculated by the 2^−△△Ct^ method. The following primers were used for qRT‐PCR analysis: lncRNA PVT1 forward, 5ʹ‐CAGCACTCTGGACGGAC‐3ʹ; lncRNA PVT1 reverse, 5ʹ‐CAACAGGAGAAGCAAACA‐3ʹ. GAPDH forward, 5ʹ‐ACTAGGCGCT CACTGTTCTC‐3ʹ; GAPDH reverse, 5ʹ‐ATCCGTTGACTCCGACCTTC‐3ʹ.

### 2.5 Western blotting

Total protein was extracted from the cells and tissues using radioimmunoprecipitation assay (RIPA) lysis buffer (Biosharp). The extracted protein was denatured using loading buffer (R&D Systems). The protein sample was subjected to sodium dodecyl sulfate‐polyacrylamide gel electrophoresis (SDS‐PAGE) using a 10% gel. The resolved proteins were transferred onto a polyvinyl difluoride (PVDF) membrane (Millipore). The membrane was incubated with the following primary antibodies (all from Abcam) at 4°C overnight: anti‐GAPDH (ab181602), anti‐E‐cadherin (ab194982), anti‐vimentin (ab92547), anti‐N‐cadherin (ab18203), and anti‐Snail (ab229701). The membrane was washed three times with Tris‐buffered saline containing Tween‐20 (TBST). The membrane was then incubated with the secondary antibody at room temperature for 1 hour. The membrane was washed three times with TBST. The protein bands were visualized using enhanced chemiluminescence (ECL) solution.

### 2.6 CCK‐8 assay

Cell proliferation was analyzed using the CCK8 kit (Dojindo, Japan). Briefly, the cells (1 × 10^5^ cells/well) were inoculated into 96‐well plates and cultured in DMEM at 37°C for 0, 24, 48, and 72 hours. Then, 10 μL of CCK‐8 solution was added to each well. The absorbance of the solution was measured at 450 nm using a microplate reader (Bio‐Rad).

### 2.7 Colony formation assay

Briefly, the cells were inoculated in a 6‐well plate (500 cells/well) and cultured in complete DMEM for 8‐12 days. The cells were washed with phosphate buffered saline (PBS), fixed in methanol for 15 minutes, and stained with 0.1% crystal violet for 15 minutes.

### 2.8 Transwell assay

Migration and invasion assays were performed using a Transwell chamber. For the invasion assay, the upper Transwell chamber was pre‐coated with Matrigel (BD Biosciences). For the migration assay, Matrigel was not added. Briefly, the cells (1 × 10^5^) were inoculated in the upper Transwell chamber in a serum‐free medium. To the lower chamber, medium containing 10% FBS was added. The cells were incubated at 37°C and 5% CO_2_. The cells were fixed in methanol for 15 minutes, stained with 0.1% crystal violet for 15 minutes, and counted under a microscope.

### 2.9 Wound healing assay

The wound healing assay was performed to evaluate the cell migration ability. Briefly, the cells were inoculated in a 6‐well plate until they formed a monolayer. Next, a scratch wound was introduced in the monolayer using a 200‐μL pipette tip. The cells were incubated in complete medium at 37°C and 5% CO_2_. The representative images were captured at 0 and 24 hours to evaluate wound healing.

### 2.10 Statistical analysis

All statistical analyses were performed using SPSS 20.0 software (SPSS Inc) and GraphPad Prism 7 (GraphPad Software Inc). The nominal data were analyzed by the chi‐scuare test, whereas the enumeration data were analyzed by the *t* test. Moreover, the Chi‐square test was used to analyze the relationship between lncRNA PVT1 expression and clinicopathological features of patients. Kaplan‐Meier and log‐rank tests were used to analyze the effects of clinicopathological characteristics on patient prognosis. In this study, the overall survival (OS) was used as the main endpoint. The difference was considered statistically significant when the *P*‐value was <.05.

## 3 RESULTS

### 3.1 Bioinformatic prediction

**FIGURE 1:**
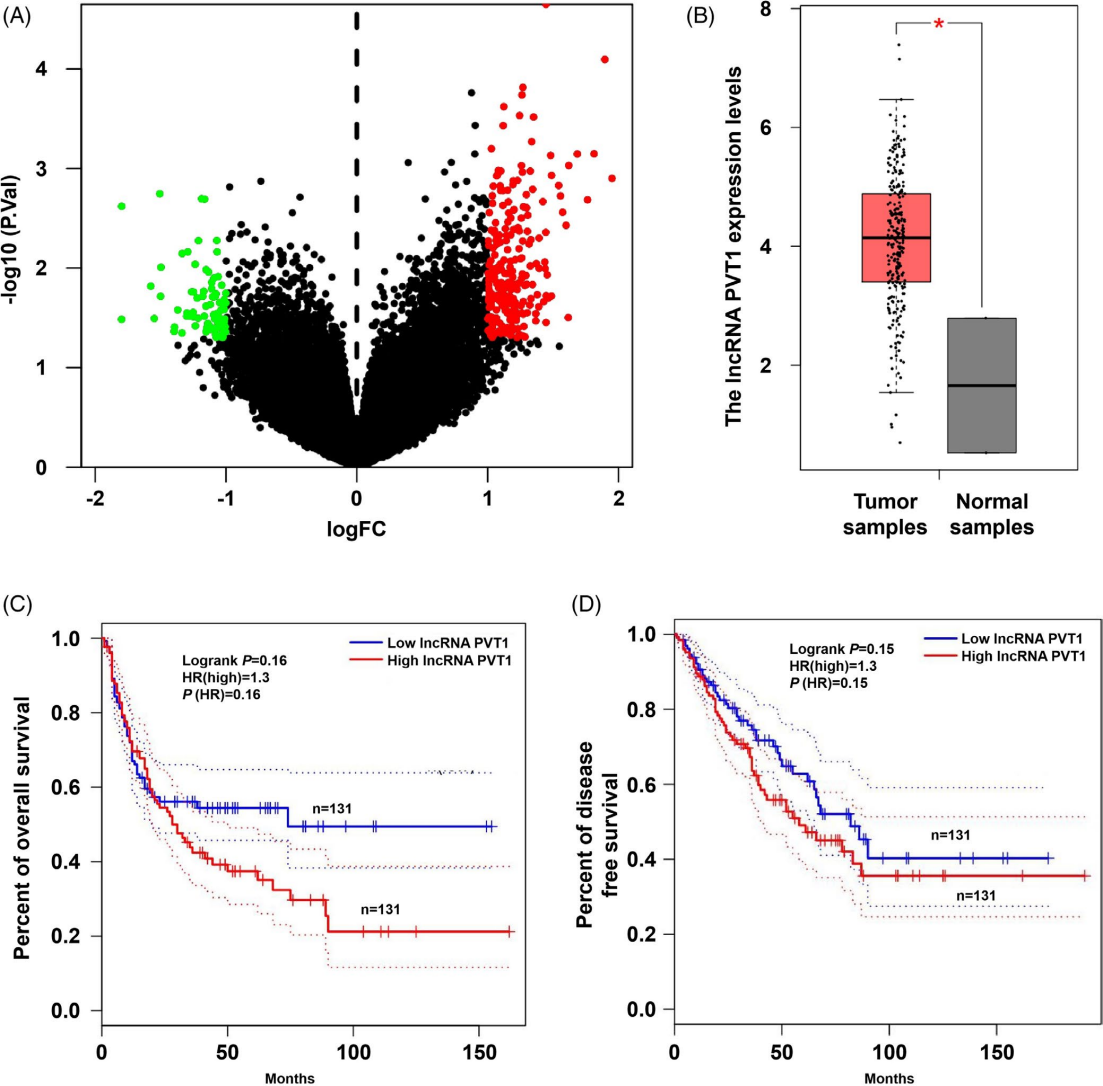
Bioinformatics analysis results. (A) Volcano map of the differentially expressed lncRNAs; (B) the expression level of lncRNA PVT1 in tumor samples is significantly higher than that of normal samples based on the prediction results of GEPIA online tools; (C) relationship between expression of lncRNA PVT1 and overall survival of tumor patients; (D) relationship between lncRNA PVT1 expression and disease free survival in tumor patients. *P < .05

We analyzed the five GeneChip microarrays of osteosarcoma and constructed a volcano plot of the differentially expressed lncRNAs (Figure 1A). In total, 237 differentially expressed lncRNAs were identified, including 57 downregulated lncRNAs and 180 upregulated lncRNAs. The expression of lncRNA PVT1 was markedly upregulated in osteosarcoma tissue. The GEPIA online analysis revealed that the osteosarcoma samples exhibited significantly higher expression of lncRNA PVT1 than that of the normal samples (Figure 1B). The survival analysis revealed that high lncRNA PVT1 expression was associated with poorer prognosis of osteosarcoma (Figure 1C,D).

### 3.2 Upregulated expression of lncRNA PVT1 in osteosarcoma cells and tissues

**FIGURE 2:**
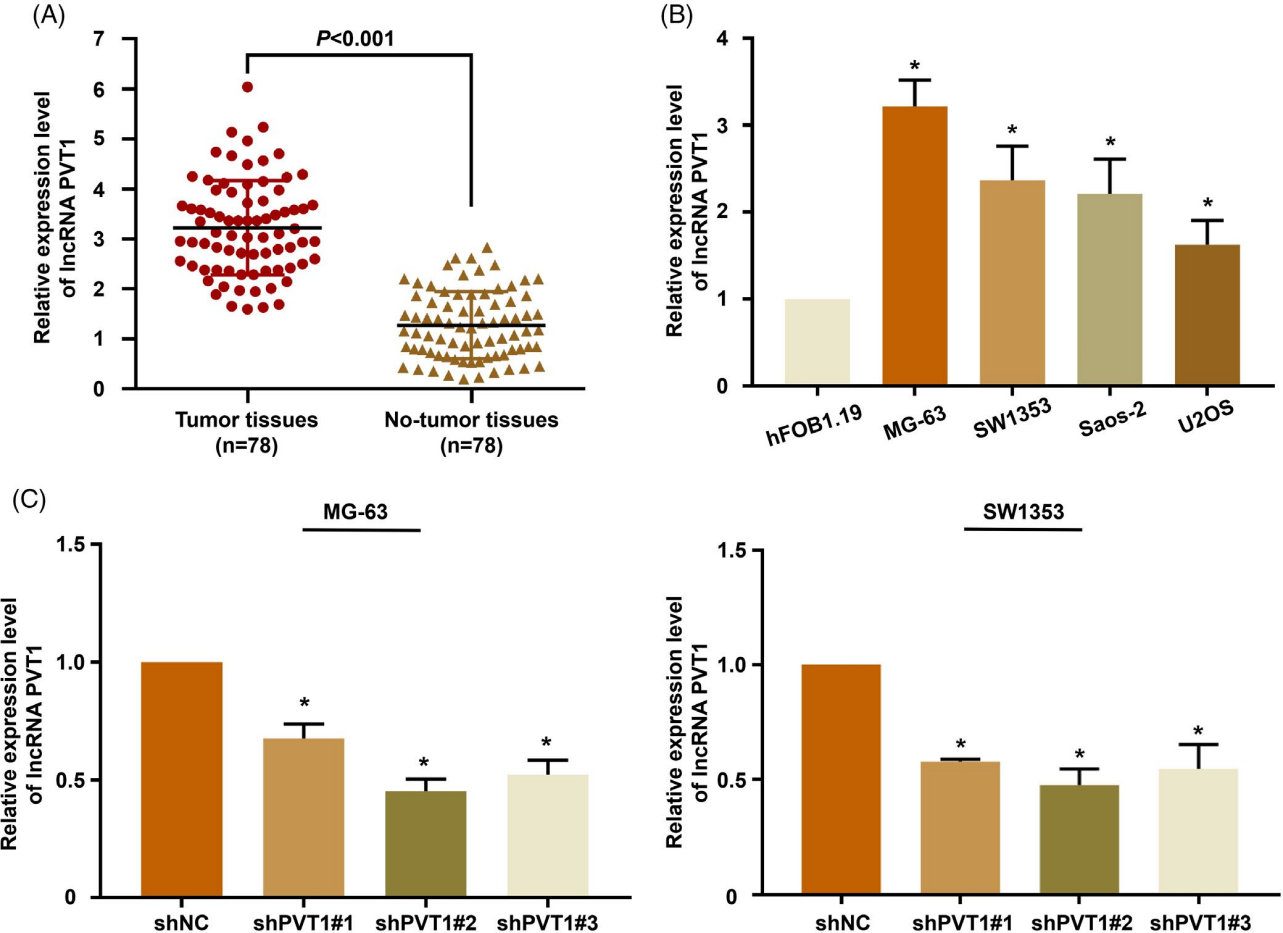
LncRNA PVT1 is upregulated in osteosarcoma. (A) The lncRNA PVT1 expression in the 78 pairs of osteosarcoma and adjacent normal tissues was detected by qRT‐PCR; (B) the expression of lncRNA PVT1 in osteosarcoma cell lines and normal human osteoblast cell lines was determined by qRT‐PCR; (C) the interference efficiency of lncRNA PVT1 expression was verified by qRT‐PCR. *P < .001

The results of the bioinformatics analysis were validated by qRT‐PCR. The expression of lncRNA PVT1 was analyzed in 78 pairs of osteosarcoma tissues and adjacent non‐tumorous tissues. The osteosarcoma tissues exhibited significantly higher lncRNA PVT1 expression than that of the adjacent non‐tumorous tissues (Figure 2A). The expression of lncRNA PVT1 was also determined in osteosarcoma cell lines. As shown in Figure 2B, the osteosarcoma cell lines exhibited higher lncRNA PVT1 expression than the normal osteoblast cell line hFOB1.19. Of the different osteosarcoma cell lines, the MG‐63 and SW1353 cell lines displayed the highest expression of lncRNA PVT1 and were selected for the subsequent cell function assays.

### 3.3 Correlation of lncRNA PVT1 expression with prognosis in patients with osteosarcoma

**FIGURE 3:**
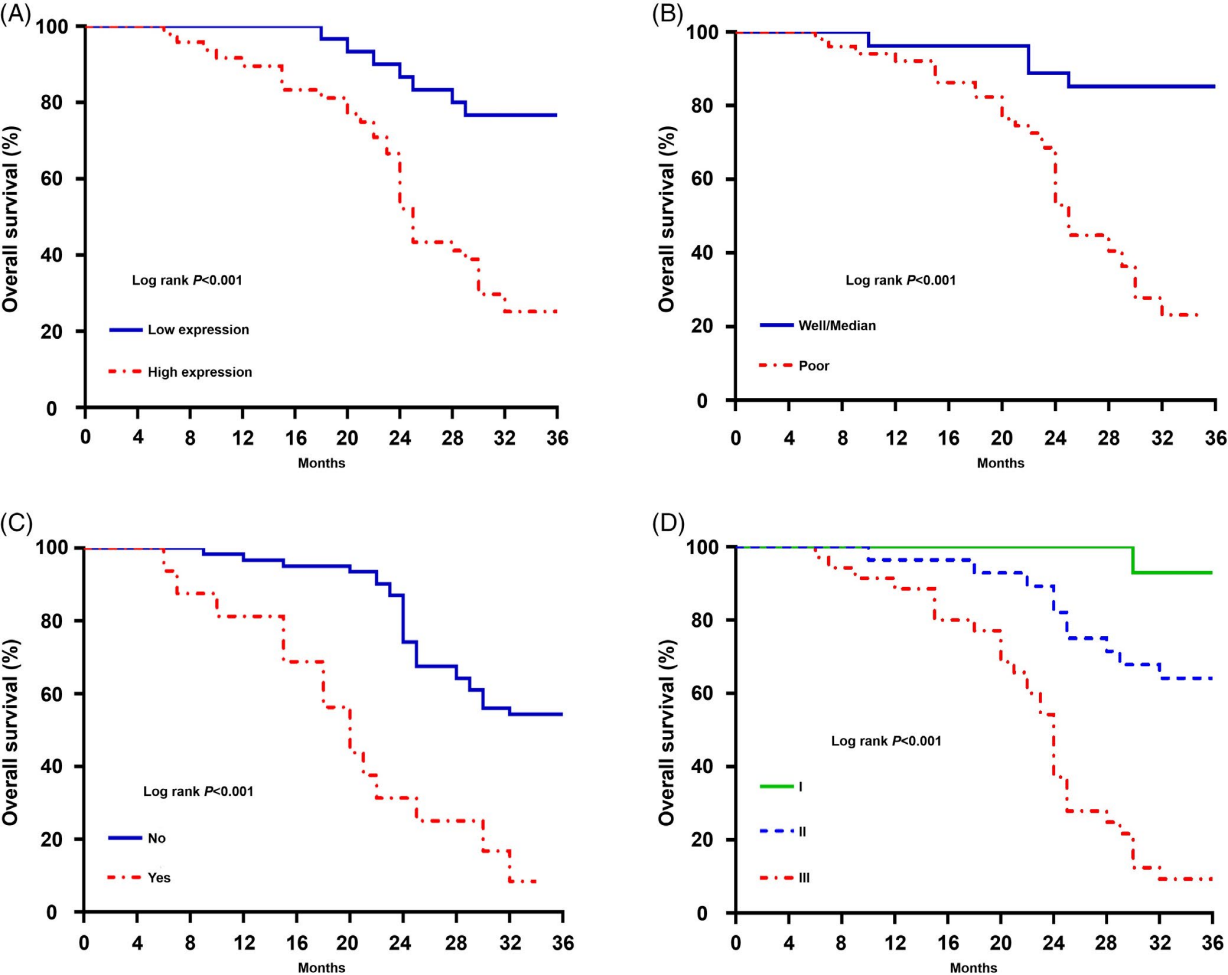
Survival curve of 78 cases patients with osteosarcoma. (A) Expression of lncRNA PVT1 and overall survival in patients with osteosarcoma; (B) degree of tumor differentiation and overall survival in patients with osteosarcoma; (C) distant metastasis and overall survival in patients with osteosarcoma; (D) disease stage and overall survival in patients with osteosarcoma

To investigate the correlation between lncRNA PVT1 expression and clinicopathological features of patients with osteosarcoma, 78 patients were categorized into the high lncRNA PVT1 (n = 48) and low lncRNA PVT1 (n = 30) expression groups using the average expression of lncRNA PVT1 as a cutoff value. The enhanced expression of lncRNA PVT1 was strongly correlated with the degree of tumor differentiation, distant metastasis, and disease stage in patients with osteosarcoma (*P* < .05; Table 1). However, lncRNA PVT1 expression was not correlated with age, sex, tumor location, tumor size, or pathological grade (Table 1). The OS of patients with osteosarcoma after follow‐up was plotted. The log‐rank test analysis revealed that the high lncRNA PVT1 expression group had a poorer OS than that of the low lncRNA PVT1 expression group (Figure 3A). In addition, the main factors affecting the OS of patients with osteosarcoma were differentiation, distant metastasis, and disease stage (Figure 3B‐D).

**TABLE 1:** Associations between LncRNA PVT1 expression and clinicopathological characteristics of patients with osteosarcoma

Characteristics	n	Low expression (n = 30)	High expression (n = 48)	*χ* ^2^	*P*
Age (y)					
<18	30	9 (30.00)	21 (43.75)	1.475	.225
≥18	48	21 (70.00)	27 (56.25)		
Gender					
Female	27	12 (40.00)	15 (31.25)	0.625	.429
Male	51	18 (60.00)	33 (68.75)		
Tumor site					
Femur/tibia	64	26 (86.67)	38 (79.17)	0.705	.401
Other	14	4 (13.33)	10 (20.83)		
Tumor size (cm)					
<3	50	18 (60.00)	32 (66.67)	0.357	.550
≥3	28	12 (40.00)	16 (33.33)		
Differentiation					
Well/Median	27	17 (56.67)	10 (20.83)	10.474	.001
Poor	51	13 (43.33)	38 (79.17)		
Histological grade					
G1‐G2	38	18 (60.00)	20 (41.67)	2.484	.115
G3‐G4	40	12 (40.00)	28 (58.33)		
Distant metastasis					
No	62	28 (93.33)	34 (70.83)	5.732	.017
Yes	16	2 (6.67)	14 (29.17)		
Disease stage					
I	15	10 (33.33)	5 (10.42)	24.111	.000
II	28	17 (56.67)	11 (22.92)		
III	35	3 (10.00)	32 (66.67)		

Cox regression analysis model was constructed to examine the effect of lncRNA PVT1 on the prognosis of patients with osteosarcoma. Univariate regression analysis revealed that tumor size, differentiation, historical grade, distant metastasis, and disease stage were highly correlated with OS in patients with osteosarcoma (Table 2). Multivariate regression analysis revealed that high expression of lncRNA PVT1 was an independent risk factor for the prognosis of patients with osteosarcoma (Table 2).

**TABLE 2:** Univariate and multivariate Cox regression of prognostic factors of patients with osteosarcoma

Parameter	Univariate analysis			Multivariate analysis		
	HR	95% Cl	*P*	HR	95% Cl	*P*
Age (≤18 y vs > 18 y)	0.791	0.429‐1.457	.452			
Gender (Female vs male)	1.663	0.851‐3.250	.137			
Tumor site (Femur/tibia vs other)	1.755	0.861‐3.575	.121			
Tumor size (<3 vs > 3 cm)	1.969	1.071‐3.622	.029	1.755	0.93‐3.314	.083
Differentiation (well/Median vs Poor)	7.881	2.793‐22.238	.000	1.759	0.896‐3.454	.101
Histological grade(G1‐G2 vs G3‐G4)	1.888	1.011‐3.523	.046	2.930	1.006‐8.529	.049
Distant metastasis (no vs yes)	4.221	2.195‐8.119	.000	2.091	1.018‐4.296	.045
Disease stage (I‐II vs III)	4.944	2.703‐9.042	.000	3.793	1.794‐8.021	.000
LncRNA PVT1 expression (Low vs high)	4.695	2.075‐10.621	.000	1.308	1.088‐3.504	.039

### 3.4 Effects of lncRNA PVT1 on the biological behavior of osteosarcoma cells

**FIGURE 4:**
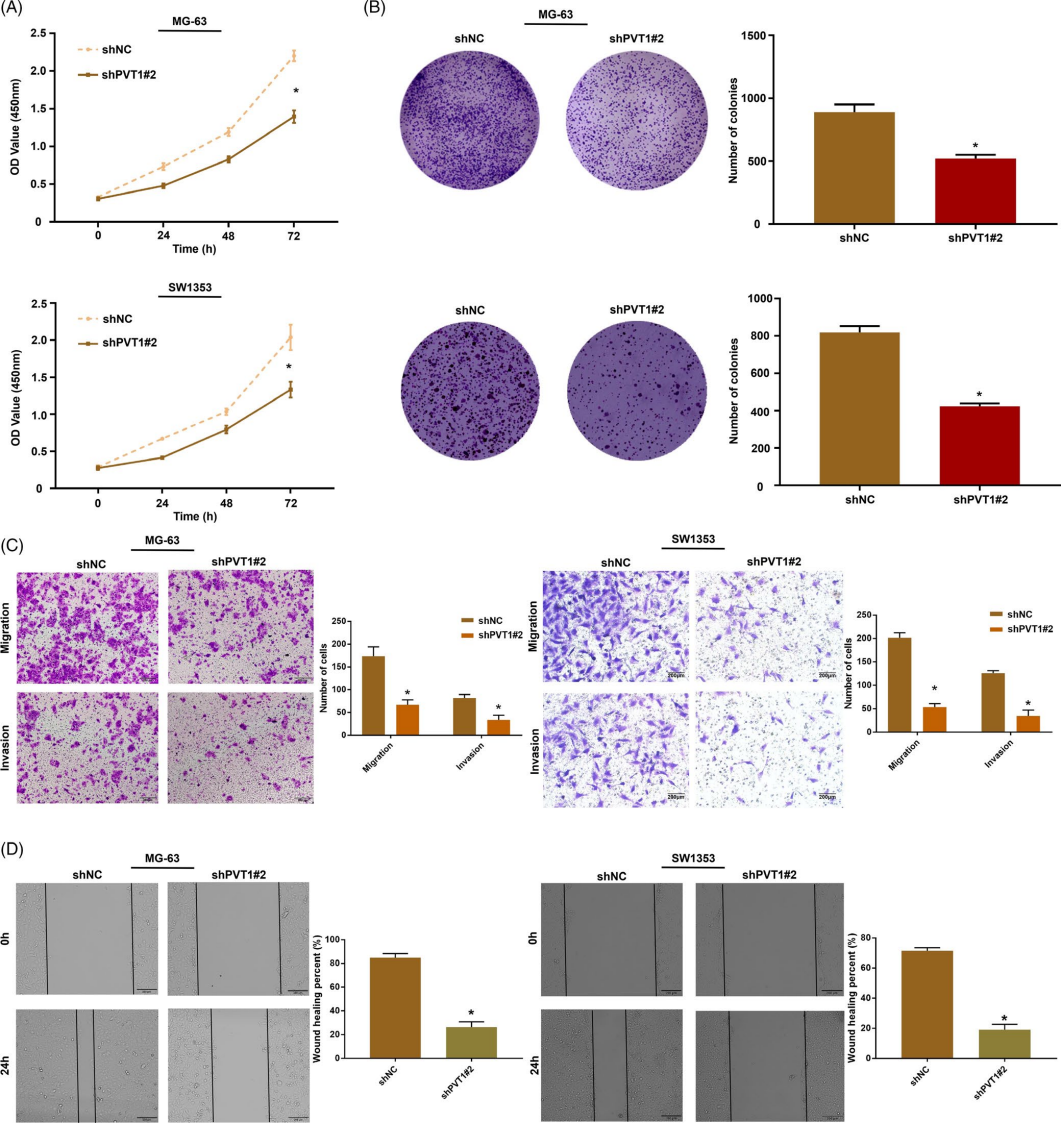
LncRNA PVT1 and malignant biological behavior of osteosarcoma cells. (A) Analysis of the effect of shPVT1 on the proliferation of osteosarcoma cells by CCK‐8; (B) the colony formation assay was used to evaluate the effect of shPVT1 on osteosarcoma cell viability; (C) detection the effect of shPVT1 on migration and invasion of osteosarcoma cells by transwell assay; (D) wound healing was used to analyze the effect of shPVT1 on the migration ability of osteosarcoma cells. *P < .001

The role of lncRNA PVT1 in osteosarcoma cells was analyzed in vitro using the MG‐63 and SW1353 cell lines. The expression of lncRNA PVT1 was silenced in MG‐63 and SW1353 cells using lentiviral transfection and validated using qRT‐PCR (Figure 2C). The effect of lncRNA PVT1 knockdown on the proliferation of osteosarcoma cells was evaluated by CCK‐8 assay. The proliferation of shPVT1‐transfected cells was significantly suppressed compared with that of shNC‐transfected cells (Figure 3A). The colony formation capacity of shPVT1‐transfected tumor cells was significantly lower than that of shNC‐transfected cells (Figure 4B). The effect of lncRNA PVT1 knockdown on the migration and invasion of osteosarcoma cells was evaluated by the Transwell assay. The knockdown of lncRNA PVT1 significantly inhibited the migration and invasion abilities of osteosarcoma cells (Figure 4B). The effect of lncRNA PVT1 knockdown on the migration of osteosarcoma cells was evaluated by wound healing assay. As shown in Figure 4D, the migration of shPVT1‐transfected cells was significantly suppressed compared with that of shNC‐transfected cells.

### 3.5 Role of lncRNA PVT1 in the EMT of osteosarcoma cells

**FIGURE 5:**
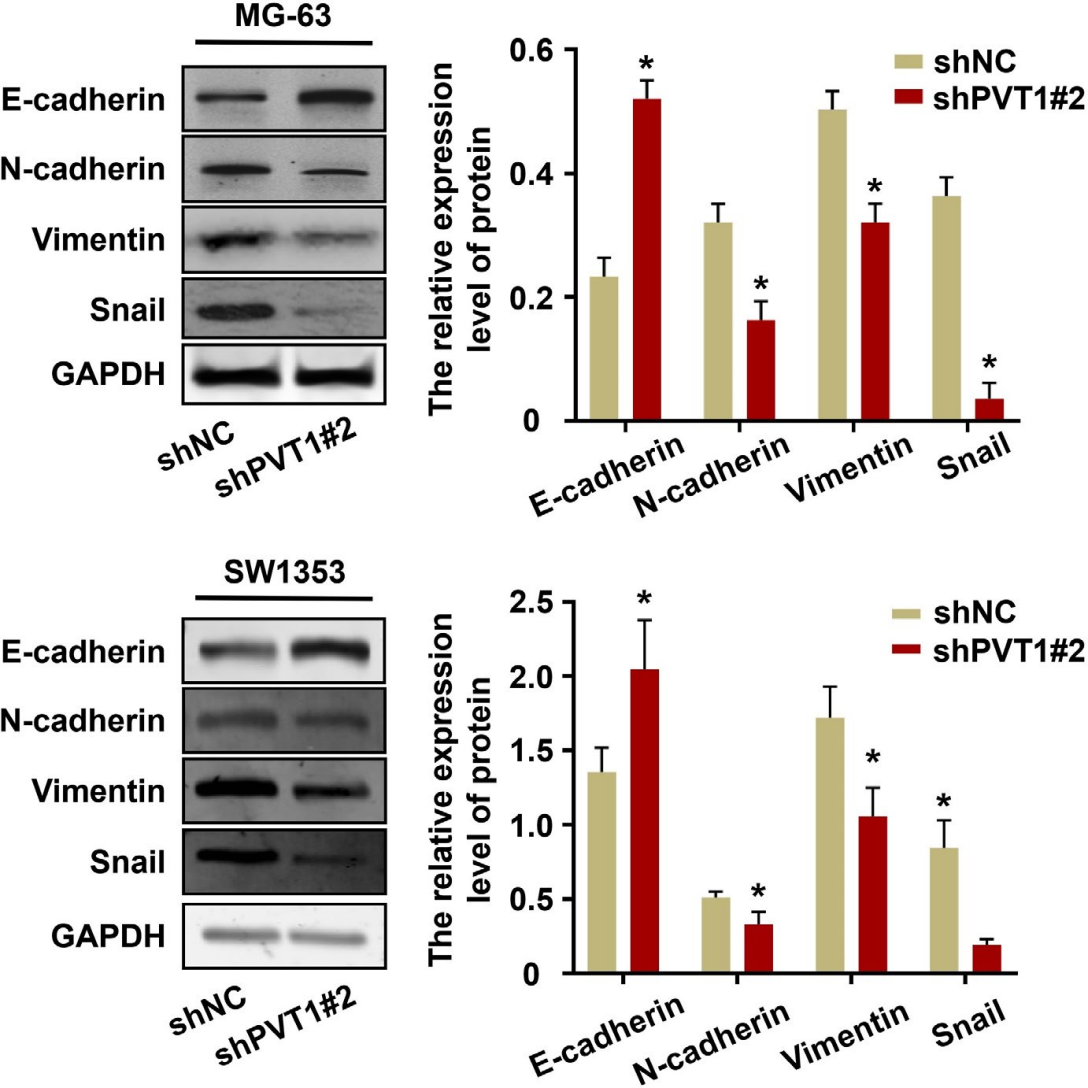
Analysis of the effect of shPVT1 on EMT of osteosarcoma cells by western blot. *P < .001

The EMT is a key step in tumor cell metastasis. Thus, the potential roles of lncRNA PVT1 in the EMT of osteosarcoma cells were analyzed. Silencing of lncRNA PVT1 affected the expression of EMT‐associated molecules in osteosarcoma MG‐63 and SW1353 cells. The lncRNA PVT1 knockdown cells exhibited enhanced expression of E‐cadherin and decreased expression of N‐cadherin, vimentin, and Snail (Figure 5).

## 4 DISCUSSION

LncRNA PVT1 is reported to be a common retrovirus integration site in mouse leukemia.^15^ The integration site is located in the sense strand of chromosome 8q24 spanning over a genomic region of 300 kb with a length of approximately 1716 nucleotides. The chromosome 8q24 region is the major target for DNA copy number amplification in tumor cells. The abnormal amplification of this region is generally predictive of a high risk of carcinogenesis.^16^ Several studies have revealed that abnormal lncRNA PVT1 expression plays a critical regulatory role in biological phenotypes, including cell proliferation, angiogenesis, cell cycle, migration, and invasion.^17^ Yang et al^18^ showed that the lung cancer tissues and cells exhibit increased lncRNA PVT1 expression, which is closely correlated with the pathological stage and lymph node involvement in patients with lung cancer. In addition, they demonstrated that patients with high lncRNA PVT1 expression have a poor prognosis and that the downregulation of lncRNA PVT1 expression can inhibit the invasion, migration, and proliferation of tumor cells. Chai et al^19^ demonstrated that the overexpression of lncRNA PVT1 promotes the invasion, migration, and proliferation of colorectal cancer cells in vitro and that lncRNA PVT1, which functions as a competitive endogenous RNA, regulates tumor growth by sponging miR‐455. Niu et al^20^ demonstrated that gastric cancer tissues exhibit significantly enhanced expression of lncRNA PVT1 and that lncRNA PVT1 can regulate the invasion and proliferation of gastric cancer cells via targeted regulation of miR‐125 activity. Zhang et al^21^ revealed that lncRNA PVT1 expression was upregulated in glioma and that silencing of lncRNA PVT1 induces G0/G1 cell cycle arrest and suppresses cell invasion and proliferation.

There are limited studies on the role of lncRNA PVT1 in osteosarcoma. In 2017, Zhou et al^22^ reported that osteosarcoma tissues overexpress lncRNA PVT1 and that silencing lncRNA PVT1 induces cell cycle arrest and apoptosis and suppresses migration, proliferation, and invasion of osteosarcoma cells by exerting a negative regulatory effect on miR‐195. Song et al^23^ reported that lncRNA PVT1 is also involved in glucose metabolism and proliferation of osteosarcoma cells through the miR‐497/HK2 pathway. Sun et al^24^ demonstrated that lncRNA PVT1 is involved in the resistance of osteosarcoma cells through activation of the c‐MET/PI3K/AKT pathway. Recently, Chen et al^25^ revealed that ALKBH5‐mediated m6A modification of lncRNA PVT1 is involved in the occurrence of osteosarcoma. Consistent with these studies, the results of this study revealed that the expression of lncRNA PVT1 was upregulated in osteosarcoma cell lines and tissues. In addition, we demonstrated that lncRNA PVT1 expression was highly correlated with TNM stage, tumor differentiation, and distant metastasis in patients with osteosarcoma. Furthermore, high expression of lncRNA PVT1 can be utilized as an independent prognostic risk indicator for osteosarcoma. Knockdown of lncRNA PVT1 expression suppressed the invasion, migration, and proliferation of osteosarcoma cells.

EMT is involved in tumor metastasis and invasion.^26^ The hallmarks of EMT are decreased expression of E‐cadherin and increased expression of vimentin and N‐cadherin.^27^ Various studies have confirmed the correlation between lncRNAs and EMT key effectors during carcinogenesis: Zheng et al^28^ revealed that lncRNA PVT1 can regulate the invasion of esophageal cancer cells by inducing EMT; Zhang et al^29^ proposed that lncRNA PVT1 may play an oncogenic role by regulating EMT through the TGFβ/Smad pathway in pancreatic cancer. In this study, we demonstrated that knockdown of lncRNA PVT1 expression could enhance E‐cadherin expression and decrease N‐cadherin and vimentin expression. These results were consistent with those of Zhang et al This indicated that lncRNA PVT1 promoted osteosarcoma by regulating EMT.

## 5 CONCLUSION

This study demonstrated the upregulation of lncRNA PVT1 expression in osteosarcoma cells and tissues and that lncRNA PVT1 is a potential therapeutic target for osteosarcoma. However, further studies are needed to elucidate the molecular mechanism by which lncRNA PVT1 promotes the progression of osteosarcoma.

## References

[1] Longhi A , Errani C , De Paolis M , et al. Primary bone osteosarcoma in the pediatric age: state of the art. Cancer Treat Rev. 2006;32(6):423‐436. 16860938 10.1016/j.ctrv.2006.05.005

[2] Mialou V , Philip T , Kalifa C , et al. Metastatic osteosarcoma at diagnosis: prognostic factors and long‐term outcome–the French pediatric experience. Cancer. 2005;104(5):1100‐1109. 16015627 10.1002/cncr.21263

[3] Wilusz JE , Sunwoo H , Spector DL . Long noncoding RNAs: functional surprises from the RNA world. Genes Dev. 2009;23(13):1494‐1504. 19571179 10.1101/gad.1800909 PMC3152381

[4] Lipovich L , Johnson R , Lin CY . MacroRNA underdogs in a microRNA world: evolutionary, regulatory, and biomedical significance of mammalian long non‐protein‐coding RNA. Biochim Biophys Acta. 2010;1799(9):597‐615. 20951849 10.1016/j.bbagrm.2010.10.001

[5] Shtivelman E , Henglein B , Groitl P , et al. Identification of a human transcription unit affected by the variant chromosomal translocations 2;8 and 8;22 of Burkitt lymphoma. Proc Natl Acad Sci USA. 1989;86(9):3257‐3260. 2470097 10.1073/pnas.86.9.3257 PMC287109

[6] Cory S , Graham M , Webb E , et al. Variant (6;15) translocations in murine plasmacytomas involve a chromosome 15 locus at least 72 kb from the c‐myc oncogene. Embo j. 1985;4(3):675‐681. 3924592 10.1002/j.1460-2075.1985.tb03682.x PMC554241

[7] Tang J , Li Y , Sang Y , et al. LncRNA PVT1 regulates triple‐negative breast cancer through KLF5/beta‐catenin signaling. Oncogene. 2018;37(34):4723‐4734. 29760406 10.1038/s41388-018-0310-4

[8] Du P , Hu C , Qin Y , et al. LncRNA PVT1 mediates antiapoptosis and 5‐fluorouracil resistance via increasing Bcl2 expression in gastric cancer. J Oncol. 2019;2019:1‐10. 10.1155/2019/9325407 PMC6530232 31205469

[9] Shang AQ , Wang W , Yang YB , et al. Knockdown of long noncoding RNA PVT1 suppresses cell proliferation and invasion of colorectal cancer via upregulation of microRNA‐214‐3p. Am J Physiol Gastrointest Liver Physiol. 2019;317(2):G222‐G232. 31125260 10.1152/ajpgi.00357.2018

[10] Cho SW , Xu J , Sun R , et al. Promoter of lncRNA gene PVT1 is a tumor‐suppressor DNA boundary element. Cell. 2018;173(6):1398‐1412. 29731168 10.1016/j.cell.2018.03.068 PMC5984165

[11] Gupta S , Maitra A . EMT: matter of life or death? Cell. 2016;164(5):840‐842. 26919422 10.1016/j.cell.2016.02.024

[12] Kalluri R , Weinberg RA . The basics of epithelial‐mesenchymal transition. J Clin Invest. 2009;119(6):1420‐1428. 19487818 10.1172/JCI39104 PMC2689101

[13] Liu W , Liu P , Gao H , Wang X , Yan M . Long non‐coding RNA PGM5‐AS1 promotes epithelial‐mesenchymal transition, invasion and metastasis of osteosarcoma cells by impairing miR‐140‐5p‐mediated FBN1 inhibition. Mol Oncol. 2020. 10.1002/1878-0261.12711 PMC7530781 32412676

[14] Yan L , Wu X , Yin X , Du F , Liu Y , Ding X . LncRNA CCAT2 promoted osteosarcoma cell proliferation and invasion. J Cell Mol Med. 2018;22(5):2592‐2599. 29502343 10.1111/jcmm.13518 PMC5908115

[15] Zeidler R , Joos S , Delecluse HJ , et al. Breakpoints of Burkitt's lymphoma t(8;22) translocations map within a distance of 300 kb downstream of MYC. Genes Chromosom Cancer. 1994;9(4):282‐287. 7519050 10.1002/gcc.2870090408

[16] Tseng Y‐Y , Bagchi A . The PVT1‐MYC duet in cancer. Mol Cell Oncol. 2015;2(2):e974467. 27308428 10.4161/23723556.2014.974467 PMC4904896

[17] Lee SC‐W , Abdel‐Wahab O . Therapeutic targeting of splicing in cancer. Nat Med. 2016;22(9):976‐986. 27603132 10.1038/nm.4165 PMC5644489

[18] Yang YR , Zang SZ , Zhong CL , et al. Increased expression of the lncRNA PVT1 promotes tumorigenesis in non‐small cell lung cancer. Int J Clin Exp Pathol. 2014;7(10):6929‐6935. 25400777 PMC4230094

[19] Chai J , Guo D , Ma W , et al. A feedback loop consisting of RUNX2/LncRNA‐PVT1/miR‐455 is involved in the progression of colorectal cancer. Am J Cancer Res. 2018;8(3):538‐550. 29637007 PMC5883102

[20] Niu J , Song X , Zhang X . Regulation of lncRNA PVT1 on miR‐125 in metastasis of gastric cancer cells. Oncol Lett. 2020;19(2):1261‐1266. 31966056 10.3892/ol.2019.11195 PMC6956415

[21] Zhang Y , Yang G , Luo Y . Long non‐coding RNA PVT1 promotes glioma cell proliferation and invasion by targeting miR‐200a. Exp Ther Med. 2019;17(2):1337‐1345. 30680011 10.3892/etm.2018.7083 PMC6327645

[22] Zhou Q , Chen F , Zhao J , et al. Long non‐coding RNA PVT1 promotes osteosarcoma development by acting as a molecular sponge to regulate miR‐195. Oncotarget. 2016;7(50):82620‐82633. 27813492 10.18632/oncotarget.13012 PMC5347719

[23] Song J , Wu X , Liu F , et al. Long non‐coding RNA PVT1 promotes glycolysis and tumor progression by regulating miR‐497/HK2 axis in osteosarcoma. Biochem Biophys Res Commun. 2017;490(2):217‐224. 28602700 10.1016/j.bbrc.2017.06.024

[24] Sun ZY , Jian YK , Zhu HY , et al. lncRNAPVT1 targets miR‐152 to enhance chemoresistance of osteosarcoma to gemcitabine through activating c‐MET/PI3K/AKT pathway. Pathol Res Pract. 2019;215(3):555‐563. 30661902 10.1016/j.prp.2018.12.013

[25] Chen S , Zhou L , Wang Y . ALKBH5‐mediated m(6)A demethylation of lncRNA PVT1 plays an oncogenic role in osteosarcoma. Cancer Cell Int. 2020;20(1):34. 32021563 10.1186/s12935-020-1105-6 PMC6993345

[26] Guo S , Xu X , Tang Y , et al. miR‐15a inhibits cell proliferation and epithelial to mesenchymal transition in pancreatic ductal adenocarcinoma by down‐regulating Bmi‐1 expression. Cancer Lett. 2014;344(1):40‐46. 24252251 10.1016/j.canlet.2013.10.009

[27] Kitamura K , Seike M , Okano T , et al. MiR‐134/487b/655 cluster regulates TGF‐β‐induced epithelial‐mesenchymal transition and drug resistance to gefitinib by targeting MAGI2 in lung adenocarcinoma cells. Mol Cancer Ther. 2014;13(2):444‐453. 24258346 10.1158/1535-7163.MCT-13-0448

[28] Zheng X , Hu H , Li S . High expression of lncRNA PVT1 promotes invasion by inducing epithelial‐to‐mesenchymal transition in esophageal cancer. Oncol Lett. 2016;12(4):2357‐2362. 27698800 10.3892/ol.2016.5026 PMC5038502

[29] Zhang X , Feng W , Zhang J , et al. Long noncoding RNA PVT1 promotes epithelialmesenchymal transition via the TGFbeta/Smad pathway in pancreatic cancer cells. Oncol Rep. 2018;40(2):1093‐1102. 29845201 10.3892/or.2018.6462

